# Clinical and financial outcomes of switching insulin glargine to insulin detemir in a veteran population with type 2 diabetes

**DOI:** 10.1186/s40200-015-0180-z

**Published:** 2015-06-26

**Authors:** Bernadette D. Asias, Eileen M. Stock, Nancy L. Small, Katerine E. Getchell, Jagruti R. Patel, Jennifer D. Krause, Staci Cavness, Cassidy L. Dzenowski, Mia Ta

**Affiliations:** Department of Pharmacy Services, Memorial Hermann Health Care System – Texas Medical Center, Houston, TX 77030 USA; Central Texas Veterans Health Care System, Department of Veterans Affairs, Temple, TX 76504 USA; Center for Applied Health Research, Central Texas Veterans Health Care System in collaboration with Baylor Scott & White Health, Temple, TX 76502 USA; Texas A&M Health Science Center, College of Medicine, Bryan, TX 77807 USA

**Keywords:** Detemir, Glargine, Glycemic control, Long-acting insulin, Type 2 diabetes, Veterans

## Abstract

**Background:**

Although glargine and detemir are both FDA-approved in the U.S. as long-acting insulin analogues, inherent differences in the insulins may lead to varying outcomes. This study examined changes in clinical measures and associated costs for veterans with type 2 diabetes on insulin therapy converted from insulin glargine to insulin detemir.

**Methods:**

A retrospective before-and-after comparison study was performed at a single-site medical center located in the southwestern U.S., comprising 133 Veterans diagnosed with type 2 diabetes receiving insulin therapy with glargine and converted to insulin detemir using a 1:1 unit dosage ratio. Patients’ A1c, weight, body mass index, total daily dose, and estimated monthly insulin costs during and after conversion were compared employing Wilcoxon signed-rank tests. These measures were similarly assessed in patients at A1c goal (<7 %) prior to conversion.

**Results:**

When switched from insulin glargine to insulin detemir, an increase in A1c (median of 7.7 % to 8.3 %, p < 0.01) and total daily dose (TDD: 40 to 46 units/day, p < 0.01) resulted. Monthly insulin costs decreased 19 % ($47 to $38, p < 0.01), or roughly a one-year savings of $110 per patient. An increase in A1c was similarly observed for patients at-goal prior to conversion but remained at-goal post-conversion (6.5 % to 6.7 %, p = 0.02).

**Conclusion:**

The increase in A1c and TDD following conversion from insulin glargine to insulin detemir suggests that glargine requires a smaller amount of units to reach the same glycemic-lowering ability compared to detemir. Despite the observed insulin cost savings associated with detemir, future studies should also determine overall costs (including indirect) and benefits associated with switching from glargine to detemir among Veteran with Type 2 diabetes.

## Introduction

In 2011, diabetes mellitus was reported to have a prevalence rate of 8.3 % in the U.S., affecting approximately 25.8 million Americans [1]. As major risk factors for diabetes, such as obesity, are becoming more common in the population, the prevalence of diabetes in adults has risen significantly from 1.9 million in 1958 to 18.8 million in 2010 [1–3]. The majority of these adults have type 2 diabetes (90-95 %) [1]. For the U.S. veteran population seeking care in the Veterans Health Administration (VA), the prevalence of diabetes is much greater. Given that approximately 20 % of this population has diabetes, there is a growing concern over the expected increase in healthcare costs associated with the rising prevalence of diabetes [1, 4].

It is estimated that individuals with diabetes have more than double the medical costs of those without diabetes [1]. This increase in costs is largely contributable to the continuous treatment of these patients, as well as the treatment of serious complications arising from diabetes, including retinopathy, nephropathy, neuropathy, and foot infections that may potentially lead to amputations. While direct medical costs associated with the treatment of diabetes in the U.S. during 2002 were estimated at $92 billion, indirect costs such as diminished productivity due to disability and mortality were estimated to be an additional $40 billion, totaling to $132 billion [5]. In 2007, the total estimated costs of diabetes rose to $174 billion, including $116 billion in direct medical costs and $58 billion from reduced productivity [6]. These estimates are overwhelming and highlight the need for greater diabetes management to improve patients’ clinical outcomes while also lowering their accrued medical costs.

Initial management of type 2 diabetes commonly entails the use of oral anti-hyperglycemics in the biguanide and sulfonylurea class. However, normal progression of the disease often leads to the need for insulin therapy for glycemic control and prevention of diabetes complications. Approximately 26 % of adults diagnosed with diabetes receive insulin therapy [1]. The use of insulin for the treatment of diabetes can have a significant impact on patients’ quality of life, as well as generate additional costs to the health care system by providing additional supplies and equipment needed for safe and effective insulin therapy management. Given the high cost associated with the management of diabetes each year and the longevity of treatment after diagnosis, these annual costs accumulated over time have immense implications for the patients themselves and the healthcare systems providing care for these patients. Long-acting insulin plays a major function in the management of diabetes due to its role in basal blood glucose control. Such agents are designed to mimic the natural physiologic effects of insulin within the human body without a defined peak in activity, thereby decreasing the risk of a hypoglycemia-related side effect. Insulin glargine and insulin detemir are currently the only FDA-approved long-acting insulin analogues available in the U.S. In clinical trials, the efficacy of insulin detemir has been shown to be equivalent to that of insulin glargine with its package labeling suggesting a 1:1 unit conversion between the two agents [7].

However, despite both glargine and detemir being classified as long-acting insulin analogues with similar efficacy, numerous studies have found substantial differences in clinical outcomes between the anti-hyperglycemic agents. For example, in several studies, insulin therapy with detemir resulted in less weight gain and a lower rate of hypoglycemia compared to therapy with glargine among patients with type 2 diabetes [8–13], suggesting that detemir may not be as potent as glargine. Isoglycemic clamp studies have shown similar duration of action between the long-acting agents at about 24 h [14]; however, the results of insulin detemir’s phase III clinical trials have shown that the two insulins vary in their duration of action [7]. Whereas insulin glargine typically has a duration of action lasting 24 h or longer, the duration of action for insulin detemir has been shown to be dose-dependent, ranging from 6 to 24 h [7, 15]. These differences may further lead to disparities in clinical outcomes for patients with type 2 diabetes prescribed either one of these insulin agents.

A recent study of 973 insulin-naïve patients initiated on either insulin glargine or insulin detemir for basal blood glucose control compared changes in A1c, the percent of patients reaching A1c goal of under 7 %, and the insulin dose required [8]. This 24-week, randomized, treat-to-target study found no difference between the insulins in A1c reduction or in the proportion of patients reaching A1c goal. However, patients receiving therapy with detemir required a significantly greater dose of insulin to achieve similar results. Patients receiving detemir were initiated on twice daily dosing, which has been associated with an increase in insulin dose without corresponding improvement in glycemic control [9]. Another randomized, treat-to-target study evaluated similar outcomes but initiated both treatment groups with once daily dosing [9]. A second dose was added to the insulin detemir group only if the pre-dinner plasma glucose levels were elevated. Similar findings were observed with no significant difference in ending A1c between the groups. However, the mean total daily dose (TDD) was lower with the use of insulin glargine.

Although clinical trials provide a well-controlled environment to compare outcomes associated insulin glargine versus insulin detemir, the stringent insulin titration protocol that is applied makes the data difficult to generalize to everyday practice. Therefore, this study aimed to examine the clinical and cost effectiveness of these insulin analogues in the context of a real-world scenario. Specifically, we assessed changes in patients’ clinical measures and medication costs associated with the conversion from insulin glargine to insulin detemir for basal blood glucose control among veterans seeking care in the VA diagnosed with type 2 diabetes. The switch from insulin glargine to detemir was due to a change in the preferred basal insulin for the institution’s formulary.

## Methods

To compare clinical and financial outcomes associated with switching from insulin glargine to insulin detemir in a veteran population with type 2 diabetes, a retrospective before-and-after study design was performed in a large integrated veteran healthcare system located in southwestern U.S. The study was approved by the local institutional review board (Central Texas Veterans Health Care System IRB) prior to study initiation. A ProClarity search of the VA National Data Cube identified 365 patients with an active prescription for both insulin detemir and insulin glargine between June 2010 and June 2011. Inclusion criteria restricted study subjects to include male veterans aged 18 to 89 years with a diagnosis of type 2 diabetes mellitus, insulin glargine treatment for a duration of at least 6 months prior to switching to insulin detemir, and a length of insulin detemir therapy for at least 6 months post-conversion. Only patients with valid clinical outcome measures collected from chart records at the time of insulin switch and post-conversion were included, reducing the study sample to 133 patients.

### Measures

Demographic measures included age and race (white, black, and other/unknown). Clinical outcome measures were assessed at the time of conversion from insulin glargine to insulin detemir (at conversion) and post-conversion (insulin therapy with detemir). Patients’ post-conversion A1c was measured at least 90 days but no more than nine months after the beginning of therapy with insulin detemir. Patients’ total daily dose (TDD, in units/day and units/kg/day), weight (kg), and body mass index (BMI, in kg/m^2^) were obtained at conversion and six months post-conversion. Patients with an A1c at goal (less than 7 %) during the insulin switch and post-conversion were documented. Oral anti-diabetic agent and pre-meal bolus short-acting insulin use at conversion was also documented. Monthly insulin costs were estimated by calculating the number of vials required to fill a 30-day supply of the insulin using TDD and the average cost per vial at the time from which dose was collected based on the local VA pharmacy invoices.

### Data analysis

Means and frequencies were calculated for patient characteristics and clinical and financial outcomes at conversion (insulin glargine) and post-conversion (insulin detemir). Analyses employing Wilcoxon signed-rank tests were performed comparing patients’ A1c, weight, BMI, and TDD, as well as estimated monthly insulin costs during and after conversion. Similar analyses were performed for patients reaching A1c goal (<7 %) prior to conversion while on insulin glargine therapy to determine if results varied. McNemar’s test was used to test marginal homogeneity for the proportion of patients observed to have at-goal (<7 %) A1c at conversion and post-conversion. Correlations between the change in A1c and both the use of oral anti-diabetic agents and pre-meal bolus short-acting insulin were assessed using Spearman’s rank correlation coefficient. All analyses were performed using SAS, Version 9.2 (Cary, NC), assuming a type I error of α = 0.05.

## Results

Among 133 male veterans switching from insulin glargine to insulin detemir, patients were predominantly older with a mean age of 66 years (SD = 10) and white (74 %; Table 1). The proportion of patients at A1c goal decreased from 18 % with insulin glargine to 16 % with insulin detemir. The proportion of veterans on pre-meal bolus short-acting insulin and oral anti-hyperglycemic agents at the time of conversion was 54 % and 59 %, respectively, with 22 % on both bolus and oral medication.

**Table 1:** Patient demographics with at conversion and post-conversion clinical measures (*N* = 133)

Patient characteristics	Frequency (%)
Age (years) Mean (SD):	66.3 (9.7)
Median (min-max):	65.0 (31.0-86.0)
Race	
White	98 (73.7)
Black	22 (16.5)
Other/Unknown	13 (9.8)
Insulin Glargine (At Conversion)	
At A1c Goal (<7 %)	24 (18.1)
Pre-Meal Bolus Insulin	72 (54.1)
Oral Anti-hyperglycemics	78 (58.7)
Both Bolus and Oral	29 (21.8)
Insulin Detemir (Post-Conversion)	
At A1c Goal (<7 %)	21 (15.8)

Following insulin conversion, patients’ A1c increased significantly from a median of 7.7 % with insulin glargine to 8.3 % with insulin detemir (p < 0.01; Table 2). This increase in A1c was observed among 61 % of patients. Similarly, the median TDD rose from 40 to 46 units/day (p < 0.01). Switching to insulin detemir therapy was associated with a change in patients’ weight with nearly two-thirds (64 %) of patients experiencing weight loss. The majority of patients (63 %) also experienced a drop in BMI (median of 31.7 to 31.5 kg/m^2^, p < 0.01). A decrease in patients’ estimated monthly insulin costs was observed during post-conversion with insulin detemir (median of $38 vs. $47, p < 0.01). That is, while TDD increased, monthly estimated insulin costs decreased 19 %. This amounts to approximately $110 per patient annually, or $11,000/year for every 100 patients. A decrease in insulin costs was observed for 74 % of the sample.

**Table 2:** Clinical and cost measures for insulin glargine at conversion versus insulin detemir post-conversion (*N* = 133)

Measures	Glargine	Detemir	p-value^*^	Frequency of trend post-conversion (%)^a^
	Mean (SD)	Mean (SD)		
	Median (Min-Max)	Median (Min-Max)		
A1c (%)	8.0 (1.4)	8.5 (1.8)	<0.01	81 (60.9) ↑
	7.7 (5.3-13.6)	8.3 (5.3-14.7)		
A1c (mmol/mol)	64 (8)	69 (4)	<0.01	81 (60.9) ↑
	61 (34–125)	67 (34–137)		
Weight (kg)	109.7 (38.9)	109.0 (37.1)	<0.01	85 (63.9) ↓
	100.0 (66.2-351.0)	100.7 (69.4-323.0)		
BMI (kg/m^2^)	32.7 (6.4)	32.3 (5.9)	<0.01	84 (63.2) ↓
	31.7 (19.3-52.3)	31.5 (21.1-49.2)		
TDD (units/day)	46.0 (29.8)	54.9 (33.6)	<0.01	79 (59.4) ↑
	40.0 (5.0-190.0)	46.0 (5.0-150.0)		
TDD (units/kg/day)	0.42 (0.2)	0.50 (0.3)	<0.01	79 (59.4) ↑
	0.39 (0.04-1.24)	0.46 (0.04-1.36)		
Estimated Cost ($)	44.24 (21.69)	40.41 (19.49)	<0.01	99 (74.4) ↓
	47.28 (21.43-141.80)	38.12 (19.06-95.30)		

^*^Wilcoxon signed-rank test

^a^Column represents number and percent of patients with particular trend for post-conversion, either increase (↑) or decrease (↓)

Although a significant increase in A1c was observed during post-conversion with insulin detemir, no difference in the proportion of patients at A1c goal at conversion versus post-conversion was observed (p = 0.47 per McNemar’s test). A weak but significant positive correlation was found between changes in A1c measurements from at conversion to post-conversion with the use of pre-meal bolus insulin (r = 0.17, p < 0.05). Bolus insulin use appeared to be associated with an increase in patients’ A1c during insulin therapy with detemir. Alternatively, the correlation between oral anti-diabetic agents and change in A1c revealed a near independent relationship (p = 0.02, p = 0.81). For patients who already achieved at A1c goal prior to insulin detemir use (*N* = 24), an increase in A1c values from a median of 6.5 % with insulin glargine to 6.7 % with insulin detemir was observed (p = 0.02; Table 3). Post-conversion, patients tend to remain at goal.

**Table 3:** Clinical and cost measures for insulin glargine at conversion versus insulin detemir post-conversion for those observed at A1c goal (<7 %) while on insulin glargine (*N* = 24)

Measures	Glargine	Detemir	p-value^*^
	Mean (SD)	Mean (SD)	
	Median (Min-Max)	Median (Min-Max)	
A1c (%)	6.3 (0.5)	7.1 (1.9)	0.02
	6.5 (5.3-6.9)	6.7 (5.3-14.5)	
A1c (mmol/mol)	45 (18)	54 (3)	0.02
	48 (34–52)	50 (34–135)	
Weight (kg)	104.9 (25.8)	104.0 (24.2)	0.27
	98.5 (68.2-169.4)	96.8 (73.1-169.9)	
BMI (kg/m^2^)	31.9 (6.7)	31.6 (6.0)	0.16
	30.0 (19.3-46.3)	29.7 (23.4-44.6)	
TDD (units/day)	36.6 (24.6)	49.4 (36.8)	0.05
	33.5(5.0-100.0)	36.0 (5.0-140.0)	
TDD (units/kg/day)	0.34 (0.2)	0.39 (0.26)	0.07
	0.33 (0.04-0.77)	0.31 (0.04-1.00)	
Estimated Cost ($)	39.13 (16.83)	33.35 (16.13)	0.02
	36.32 (23.64-70.92)	38.12 (19.06-76.12)	

^*^Wilcoxon signed-rank test

## Discussion

The results of this study reflect the outcomes of an older male veteran population diagnosed with type 2 diabetes. Although our sample size was relatively small, a significant increase in A1c was observed after switching to insulin detemir from insulin glargine. Since patients were switched using a 1:1 unit conversion, the increase in A1c suggests that insulin detemir and insulin glargine are not equipotent. In fact, based on the estimates for total daily dose between the groups, insulin glargine requires 16 % less units than insulin detemir to achieve the same glycemic control. Additionally, when examining patients who had an at goal A1c prior to the conversion to detemir, an increase in A1c was similarly observed post-conversion, though still remained at goal. Recent literature has similarly shown an insulin detemir dose requires a larger number of units compared to insulin glargine in order to reach comparable A1c levels [8–11].

Concurring with previous studies, the majority of patients experienced a decrease in weight and BMI after conversion from insulin glargine to insulin detemir. Our findings were similar to that of a recent study by Bryant and colleagues (2013), which examined 31 patients with type 1 or type 2 diabetes who converted from insulin glargine to insulin detemir after a Medicaid formulary switch [16]. Among those with type 2 diabetes, the mean basal insulin dose was higher with detemir (74 vs. 56 units/day, p < 0.01). This amounts to a requirement of 32 % higher doses of insulin detemir compared to insulin glargine. Despite insulin dose titrations, no change in A1c occurred from baseline to 12 months post-conversion, further suggesting that insulin glargine and insulin detemir are not equipotent.

In our study, the presence of pre-prandial bolus insulin and oral anti-hyperglycemics was evaluated since titration of these agents may also affect A1c levels. The presence of pre-prandial bolus insulin was weakly correlated with an increase in A1c, which is contrary to what would be expected given that the bolus insulin may also be titrated to contribute to improved glycemic control. A possible explanation for this result is that the patients on pre-prandial bolus insulin may have diabetes that is more difficult to control. The relationship between pre-meal bolus insulin use and increased A1c should be evaluated in a larger sample. Oral anti-hyperglycemics and change in A1c were found to be independent. This is likely due to the fact that most patients have been titrated up to the maximum doses of oral anti-hyperglycemics prior to initiating insulin, so titrations after the conversion were rare.

In a previous retrospective cohort analysis by Borah and colleagues (2009), insulin glargine and insulin detemir were associated with comparable mean adjusted all-cause pharmacy costs, medical costs, and total health care costs. However, insulin glargine was associated with significantly higher diabetes-related medical costs and total diabetes-related health care costs [17]. Cost estimation in this study was solely based on medication costs, and despite the increased number of units needed to achieve a similar A1c goal, a 19 % monthly cost savings results from switching insulins.

To our knowledge, this study is one of few that compare clinical outcomes and medication costs for patients with type 2 diabetes converted from insulin glargine to insulin detemir indicated for basal blood glucose control reflecting real-world results. In this study, the providers do not adhere to a strict protocol (such as with clinical trials), and practice may vary from provider to provider. Per study design, patients were compared to themselves to help control for possible confounders that may influence results. Additionally, the precise number of units the patients were using was obtained from chart review and used in the analyses. This strengthens our study as many previous retrospective studies have used dispensing histories to evaluate daily dose which may not be an accurate estimate of insulin use as these records do not account for discarded insulin [17, 18].

### Limitations

Given the sample’s size and patient characteristics, findings may be difficult to generalize to non-VA populations. Although this study reflects “real-world” results of a conversion from insulin glargine to insulin detemir, there are many confounders including change in diet, exercise, and compliance that were unadjusted for and may have affected results. Desirable future studies would entail a larger sample size with an additional group that would receive continued prescribing of insulin glargine over the entire period of the study.

Though limited by this study’s sample size, further subanalyses should compare outcomes across groups with varying BMI. When examining the subgroup of patients with A1c at-goal (<7 %, *n* = 24 patients) on glargine, the change in weight and BMI was no longer significant, and the effect of switching insulin on TDD and costs was lessened. From this, it appears that the inclusion of patients with higher baseline A1c (not at-goal) while on insulin glargine tend to initially have higher BMI, TDD, and costs. In fact, a three-fold increase in median A1c after switching insulin was observed after including patients with higher A1c (+0.2 % at-goal vs. +0.6 % all patients). Similarly, median TDD was doubled (+2.5 units/day at-goal vs. +6.0 units/day all patients). And, interestingly a median decrease in costs was observed that was five times the increase in costs observed for patients with A1c < 7 % (+$2.18 at-goal vs. – $9.16 all patients).

Initially the conversion was done on a 1:1 basis with dose titrations allowed after the conversion in order to maintain or improve the patient’s hemoglobin A1c, as needed. This led to different maximum TDD for each insulin. However, we believe the effect is minimal given that the higher maximum was observed with glargine but the average and median TDD was greater with detemir. Doses exceeding 100 units/day were typically divided equally into twice daily.

Evaluating the number of patients who required an increase in frequency of administration to twice a day dosing after conversion would help determine the actual clinical impact of the proposed dose-dependent differences in duration of action of insulin detemir. Finally, the calculation of cost only included the cost of the insulin. Other factors that may affect the cost savings seen in this study are the cost of labor required to convert patients and the cost of the additional insulin syringes for those patients who are eventually changed to twice daily dosing after the conversion. Additionally, patient indirect costs were not factored into this cost measure.

## Conclusion

In an older, mainly white veteran population with type 2 diabetes, conversion from insulin glargine to insulin detemir resulted in an increase in A1c post-conversion, decrease in weight and BMI, and an increase in total daily dose. Therefore, the study suggests that insulin glargine and insulin detemir are not equipotent. However, no difference in the proportion of patients reaching A1c goal was observed. A monthly cost savings was observed for most patients switching to insulin detemir. On average, switching to insulin detemir resulted in a 19 % reduction in costs, or per-patient savings of $110 annually on insulin expenditures.
